# Quantitative Analysis of Musculoskeletal Ultrasound: Techniques and Clinical Applications

**DOI:** 10.1155/2017/9694316

**Published:** 2017-04-30

**Authors:** Qing Wang, Qing-Hua Huang, John T. W. Yeow, Mark R. Pickering, Simo Saarakkala

**Affiliations:** ^1^Institute of Medical Information, School of Biomedical Engineering, Southern Medical University, Guangzhou, China; ^2^South China University of Technology, Guangzhou, China; ^3^University of Waterloo, Waterloo, ON, Canada; ^4^The University of New South Wales, Canberra, ACT, Australia; ^5^University of Oulu, Oulu, Finland

Musculoskeletal ultrasound is one of a number of musculoskeletal imaging modalities. It can be defined as an ultrasound imaging technique for the diagnosis and treatment of patients with musculoskeletal disorders. In comparison with advanced imaging modalities such as CT and MRI, advantages of ultrasound include the readily available bedside ultrasound equipment, the relatively low cost of the exam procedure, the capacity for dynamic evaluation of patients, and the use of nonionizing radiation. Therefore, musculoskeletal ultrasound has been widely adopted for many clinical applications. Recent advances in musculoskeletal ultrasound have provided physicians with valuable information in application areas such as sports medicine, osteoarthritis, osteoporosis, musculoskeletal pain, and rehabilitation. The new findings in these areas have also inspired novel signal and image processing techniques that are developed to facilitate many potential new clinical applications. Recently developed quantitative ultrasound techniques are able to provide quantitative assessment of the structure and function of musculoskeletal tissues. In addition, ultrasound biomicroscopy imaging with high-frequency probes is able to resolve finer imaging details of musculoskeletal tissues. In this issue readers will find 12 high-quality, peer-reviewed articles that provide researchers from diverse backgrounds such as biomedical engineering, medical ultrasound, rehabilitation, and computational sciences with the state-of-the-art knowledge of this emerging interdisciplinary research area.

In the review paper “A Review on Real-Time 3D Ultrasound Imaging Technology” by the special issue editor Q. Huang and Z. Zeng, a comprehensive review on traditionally designed 3D ultrasound techniques as well as the most advanced real-time 3D imaging using parallel computing hardware and methods is provided. It summarizes the pros and cons of different data acquisition protocols, discusses the algorithms of volume reconstruction, and introduces the clinical applications including musculoskeletal examinations.

Ultrasound images of the musculoskeletal system provide visible information of the structure of musculoskeletal tissues and organs such as muscles, tendons, ligaments, joints, and soft tissue. Ultrasonography is a useful tool to guide the treatment of patients with musculoskeletal disorders. Ultrasound-guided techniques help physicians accurately inject treatment drugs into the target tissues to satisfy the requirements of a successful and safe therapy. In this special issue, two papers introduce ultrasound-guided techniques that are applied in the treatment of musculoskeletal pain. The paper “Ultrasound-Guided versus Fluoroscopy-Guided Deep Cervical Plexus Block for the Treatment of Cervicogenic Headache” by Q. Wan et al. presents an ultrasound-guided approach to treating cervicogenic headache through injection in deep cervical plexus block. Q. Wan et al. also introduce an application of ultrasonography to guide the lumbar periradicular injection for patients with unilateral lower lumbar radicular pain in their paper entitled “Ultrasonography-Guided Lumbar Periradicular Injections for Unilateral Radicular Pain.” The ultrasound-guided approach is shown to be a potentially promising method for the pain treatment due to its convenience and efficacy.

Rehabilitation for musculoskeletal disorders needs accurate diagnosis and follow-up evaluation of the outcomes during a relative long-term physical exercise therapy procedure. In this special issue, there are four papers that report the applications of musculoskeletal ultrasound in rehabilitation. In “Ultrasonographic Validation of Anatomical Landmarks for Localization of the Tendon of the Long Head of Biceps Brachii,” S. Hou et al. introduce two anatomical landmarks for localization of biceps tendon groove and validate the localization with ultrasonographic measurement. The reliable location of treatment and monitoring plays an important role in therapy and diagnosis for patients with musculoskeletal disorders. L. Li and R. K. Tong introduce a combined method of ultrasound imaging and biomechanical modeling for post-stroke muscular evaluation in their paper entitled “Combined Ultrasound Imaging and Biomechanical Modeling to Estimate Triceps Brachii Musculotendon Changes in Stroke Survivors.” Based on the ultrasonic parameters, the subject-specific biomechanical model could predict the changes of musculotendon properties for patients after stroke. In the paper “Ultrasonic Measurement of Dynamic Muscle Behavior for Poststroke Hemiparetic Gait” by X. Chen et al., the authors developed a synchronized system for simultaneously collecting ultrasonography signals, EMG, and joint angle to dynamically analyze the functional and morphological changes during muscle contraction. Ultrasonography can be deemed as an alternative method for examination of the morphological changes for stroke patients. C.-Z. Wang et al. describe a novel ultrasound technique called “vibroultrasound” that achieves the active stiffness of the muscles under isometric contraction. Their paper entitled “Age and Sex Effects on the Active Stiffness of Vastus Intermedius under Isometric Contraction” investigates the relationship between muscle stiffness and the contraction intensity and provides insight into the age and sex bias in musculoskeletal studies.

Quantitative ultrasound, a recently developed promising method, has been applied to quantitatively assess the structure and function of musculoskeletal tissues. This special issue presents three quantitative ultrasound studies for evaluation of the tissues in musculoskeletal diseases. The paper “Early Detection of Tibial Cartilage Degradation and Cancellous Bone Loss in an Ovariectomized Rat Model” by Y. Wang et al. proposes a quantitative high-frequency ultrasound approach for the early detection of rat tibial cartilage degradation. This is a promising method to detect the early degradation of articular cartilage induced by estrogen reduction in female osteoarthritis. T. Suzuki et al. present a semiquantitative evaluation of polymyalgia rheumatica and elderly-onset rheumatoid arthritis by power Doppler ultrasound in their paper entitled “Semiquantitative Evaluation of Extrasynovial Soft Tissue Inflammation in the Shoulders of Patients with Polymyalgia Rheumatica and Elderly-Onset Rheumatoid Arthritis by Power Doppler Ultrasound.” It is indicated that power Doppler ultrasound can be a reliable tool to semiquantitatively evaluate the inflammation of soft tissues. The paper “Transverse and Oblique Long Bone Fracture Evaluation by Low Order Ultrasonic Guided Waves: A Simulation Study” by Y. Li et al. focuses on using ultrasound-guided waves to evaluate bone fracture and monitor its healing. This simulated study shows that the ultrasound-guided waves have good potential for evaluation of bone fractures and their healing monitoring.

There are two papers that report novel techniques of signal and image processing to extract and analyze the target tissues in this special issue. The paper “A Novel Segmentation Approach Combining Region- and Edge-Based Information for Ultrasound Images” by Y. Luo et al. demonstrates a new segmentation algorithm based on an improved machine learning strategy for extracting lesions from ultrasound images, for example, the breast and musculoskeletal ultrasound images. With accurate extraction of the lesion region in ultrasound images, computer aided diagnosis, which is of great help in routine musculoskeletal examinations in a clinical setting, can be easily conducted. The paper “Semianalytical Solution for the Deformation of an Elastic Layer under an Axisymmetrically Distributed Power-Form Load: Application to Fluid-Jet-Induced Indentation of Biological Soft Tissues” by M. Lu et al. presents an ultrasound indentation method for analyzing the biomechanical property of biological soft tissues. The clearer analysis of the biomechanical properties provided by this technique would be of great help in better understanding the function of the musculoskeletal tissues.

In summary, the 12 papers in the special issue report not only new clinical studies of musculoskeletal ultrasound but also recently developed ultrasound techniques for musculoskeletal tissue characterizations. We hope that this special issue will help to promote further development of musculoskeletal ultrasound methodologies for various clinical applications. Improvement and advances in musculoskeletal ultrasound may lead to better performance outcomes in diagnosis and treatment of patients with musculoskeletal disorders. Musculoskeletal ultrasound provides quantitative evaluation and dynamic monitoring and thus reduces the cost, duration, and overall impact of musculoskeletal diseases.

